# Gut Microbiota Divergence Between Wild Alpine Musk Deer and Ex-Situ Conserved Forest Musk Deer: Insights for Ex-Situ Conservation and Reintroduction Strategies

**DOI:** 10.3390/microorganisms14102309

**Published:** 2026-10-10

**Authors:** Haonan Zhang, Yichen Wang, Xianna Lan, Zhengwei Luo, Jin Bai, Liping Yan, Xinghu Qin, Defu Hu

**Affiliations:** 1 School of Ecology and Nature Conservation, Beijing Forestry University, Beijing 100083, China; 2 Banbar County Xiangrui Poverty Alleviation and Development Investment Co., Ltd., Changdu 855500, China

**Keywords:** 16S rDNA high-throughput sequencing, plateau, wild alpine musk deer, ex situ forest musk deer, gut microbiota

## Abstract

The gut microbial ecosystem is structured by the gut microbiota residing in animal hosts. It plays a critical role in modulating core host physiological functions, covering food digestion, nutrient absorption, energy metabolism and immune regulation. In this study, we performed 16S rDNA high-throughput sequencing to characterize the gut microbiota of wild alpine musk deer (*Moschus chrysogaster*, AMD) and ex situ conserved forest musk deer (*Moschus berezovskii*, FMD). We then inferred the putative functional profiles based on known taxon–function relationships. Our analyses revealed no significant difference in gut microbiota alpha diversity between wild AMD and ex situ conserved FMD. However, significant divergence in gut microbiota beta diversity was detected between the two species. The family *Muribaculaceae* and its unclassified affiliated genus as well as the genus *Monoglobus* and *Pseudomonas* were significantly more abundant in wild AMD than in ex situ conserved FMD. By contrast, the genera Prevotellaceae_UCG_004, *Alistipes*, Christensenellaceae_R-7_group, *Akkermansia* and the family *Oscillospiraceae* were significantly more abundant in ex situ conserved FMD than in wild AMD. Furthermore, the *Firmicutes*/*Bacteroidetes* ratio was numerically higher in FMD than in AMD. Together, these results indicate that the gut microbiota differences between the two musk deer species are consistent with the dietary factors differences. These findings further suggest that the formulated concentrate diet fed to FMD was lower in fiber and higher in digestible energy than the natural diet of AMD. These dietary factors may partly underlie the observed microbiota differences. Accordingly, we recommend adjusting the feeding regime and husbandry practices for FMD to better match the native food plants consumed by AMD. Such adjustment represents an evidence-based strategy for improving the health of FMD populations.

## 1. Introduction

The animal gut microbiota, composed of abundant and diverse microbial taxa, assembles into a complex microecosystem that mediates key host physiological processes. It is critical for supporting host nutritional fitness and adaptive potential [1]. Herbivores rely on symbiotic gut microbiota to degrade recalcitrant dietary polysaccharides (e.g., cellulose, hemicellulose); the composition and diversity of these communities serve as critical biomarkers for assessing host nutritional status and ecological adaptability [2,3]. Mounting evidence confirms that animal gut microbiota exhibit strong host taxonomic specificity [4]. Host intrinsic traits (e.g., age, sex) and external environmental conditions are confirmed regulators of gut microbial profiles [5,6,7]. However diet exerts a dominant effect over other drivers, and is widely recognized as the primary driver of gut microbial divergence even between closely related host species [8,9,10]. Furthermore, despite compositional divergence between the ruminal and fecal microbiotas, the abundances of core cellulose- and hemicellulose-degrading taxa are significantly correlated across these compartments, supporting the use of rumen microbiota data as a proxy for inferring fecal microbiota function.

*Moschus* (musk deer) are small forest-dwelling ungulates endemic to Asia, with native ranges spanning China and adjacent regions of northern South Asia and northern Southeast Asia. Musk, a high-value bioactive secretion, is produced by the preputial glands of sexually mature males. Musk is widely used in the global luxury perfumery industry and traditional Chinese medicine. Over the past 40 years, unsustainable harvesting, illegal poaching, and habitat loss have driven severe range contractions and wild population declines of *Moschus* spp. across China. Consequently, all *Moschus* species are listed as endangered or vulnerable taxa on the IUCN Red List of Threatened Species, and are classified as Class I State Key Protected Wild Animals in China. The vast elevational transition zone along the eastern margin of the Qinghai–Tibet Plateau (QTP) is a core sympatric distribution region for the FMD and AMD. FMD naturally occupies elevations of 500–2500 m, while AMD is endemic to higher elevations of 2200–4200 m, creating a natural sympatric overlap zone of 2200–2500 m within this transition zone. Both FMD and AMD are typical browsers. Their gut microbiota is a well-recognized biological indicator of habitat adaptation and nutritional status. The mid-to-high elevational zones of this QTP eastern margin transition zone support relatively intact forest ecosystems with low anthropogenic disturbance. Developing eco-compatible industries in this region is a core priority for advancing local socioeconomic development [11]. Since 2018, ex situ captive breeding programs for lowland-origin FMD populations have been implemented in the mid-to-high elevational zones of this transition zone. These programs have two core objectives: (1) to produce genetically viable founder individuals for wild FMD population reinforcement and reintroduction initiatives; and (2) to establish sustainable, legally compliant captive populations to support regulated, traceable musk harvesting.

FMD and AMD are closely related congeneric musk deer species, with a high degree of similarity in habitat preferences, physiological traits, and gut microbiota composition. Jiang et al. performed a longitudinal assessment of gut microbiota dynamics in ex situ conserved FMD populations across China [12]. However, site-specific differences in environmental conditions and dietary regimes are major drivers of gut microbial compositional variation, even among conspecific FMD populations [7]. Existing studies have separately assessed the gut microbiota of FMD and AMD through pairwise comparisons of taxonomic composition and functional repertoire between captive and wild conspecifics of each species. However, these studies relied on geographically disjunct sampling. They lumped co-varying variables (e.g., ambient temperature and plant resource availability) into a broad, poorly defined category of “environmental factors”. No study to date has quantified the independent contributions of these variables to gut microbial variation [13,14]. To date, direct interspecific comparative analyses of gut microbiota between sympatric FMD and AMD populations remain particularly scarce. The relevant studies reported the only analysis confirming taxonomic divergence in gut microbiota between allopatric captive FMD and AMD populations [12,15]. However, their analysis shares the core methodological limitation of all prior *Moschus* gut microbiota research: both target species were sampled from geographically isolated sites, without controlling for cross-site environmental heterogeneity. This uncontrolled variation is a major confounder in interspecific comparisons. Consequently, observed microbial differences among populations cannot be reliably attributed to host taxonomic identity, as they may instead reflect site-specific environmental effects. To address this long-standing confounding bias and fill critical gaps in microbial research pertaining to musk deer conservation, this study aimed to achieve two objectives: (1) to develop a controlled sympatric sampling framework that minimizes environmental confounders, and to apply it to quantify gut microbiota differences between wild AMD and ex situ conserved FMD populations; and (2) to identify the core factors driving these interspecific differences, and to refine and standardize husbandry protocols for FMD translocated to high-altitude habitats. The resulting data will support the adaptive management of gut microbial communities prior to reintroduction.

## 2. Materials and Methods

### 2.1. Study Area

This study was conducted in Nim Township, Banbar County, northwestern Qamdo City, Tibet Autonomous Region, China (31°8′28″ N, 94°18′39″ E). This region is characterized by continuous towering mountains, with a mean altitude exceeding 4000 m, and a temperate, semi-humid, plateau climate. Mean monthly temperature varies between 13 and 15 °C in July and between −15 and −5 °C in January; temperatures are low year-round, with large diurnal fluctuations. Strong winds prevail in winter and spring, while hailstorms are common in summer and autumn. Mean annual precipitation is 300–450 mm, the majority of which falls from June to September. Vegetation exhibits clear elevational zonation, with community types shifting along elevation gradients, including valley sclerophyllous broadleaved forests, mixed coniferous–broadleaved forests, pure coniferous forests, and alpine meadows. Dominant canopy tree species include *Betula platyphylla*, *Salix matsudana*, and *Hippophae rhamnoides*; dominant shrub species include *Rhododendron simsii*, *Lonicera trichosantha*, and *Elsholtzia stauntonii*. Common medium-to-large mammalian species in the region include *Panthera uncia*, *Ursus arctos*, AMD, *Capricornis milneedwardsii*, and *Cervus albirostris*.

### 2.2. Sample Collection

Fecal sampling was used as a non-invasive approach to characterize the gut microbiota. Wild AMD fecal samples were collected from 1 to 15 September 2023 in the largest mixed coniferous–broadleaved forest of the study area (elevation range: 3700–4200 m). Twelve vertical transect lines (total length 72.35 km) were established with the assistance of local Tibetan guides. Sampling sites were established along each transect (≥2 km apart), and only the freshest fecal sample at each site was retained for analysis. Samples were collected from the center of the fecal pellets, containing mucus, and transferred into 2 mL sterile centrifuge tubes. The date, coordinates, and elevation of each sampling location were recorded [15,16]. In total, 170 suspected ungulate fecal samples were collected. Fecal samples were initially preserved at −20 °C for 48 h then transported to the laboratory on dry ice, where they were stored at −80 °C until DNA extraction. Fecal material (3 mL) was vortexed with 5 mL phosphate-buffered saline (PBS) for 2 min. DNA was extracted using a TIANamp Genomic DNA Kit (Tiangen Biotech Co., Ltd., Beijing, China) according to the manufacturer’s instructions, checked by 1% agarose gel electrophoresis, and stored at −20 °C. A ~550 bp l fragment of the mitochondria 16S rDNA was amplified using primers 16S-F/R in 25 µL reactions (3 µL DNA, 12.5 µL 2× Premix Taq, 1 µL each 10 µM primer, 3.5 µL ddH_2_O, 4 µL BSA [20 µg/µL, Solarbio, Beijing, China]). PCR conditions were: initial denaturation at 95 °C for 5 min; 30 cycles of 94 °C for 30 s, 49 °C for 30 s, and 72 °C for 45 s; and a final extension at 72 °C for 5 min. Products were Sanger-sequenced (ABI 3730XL, SinoGenoMax, Beijing, China), trimmed to ~126 bp (Geneious Prime 2022.0.1), and compared against the GenBank database (NCBI: https://www.ncbi.nlm.nih.gov/) using BLAST (https://blast.ncbi.nlm.nih.gov/Blast.cgi). Species were assigned based on the top hit (100% coverage, ≥98% similarity). Species identity of all samples was verified via DNA sequencing: non-target samples from *Capricornis milneedwardsii* (mainland serow) *and Cervus albirostris* (white-lipped deer) were excluded, and only samples confirmed to originate from wild AMD were retained. In total, 35 of the 170 samples were confirmed as AMD. From these, a final set of 12 high-quality AMD fecal samples was used for downstream analyses.

Fecal samples of ex situ conserved FMD were collected on 15 September 2023 at an FMD breeding base located at 3700 m. Each FMD was housed individually in a separate pen. The FMD basal diet consisted of mixed dry foliage, fresh native foliage, and small amounts of herbaceous plants, supplemented with a mixed concentrate of succulent feeds (e.g., carrot, apple) and energy-dense ingredients (e.g., maize, peanut, etc.). All individuals had ad libitum access to clean drinking water. A total of 86 adult FMD were translocated for ex situ conservation, including 27 males from these, 9 healthy males were selected for fecal sampling.

### 2.3. DNA Extraction and Sequencing of 16S rRNA Gene Amplicons

Total microbial genomic DNA was extracted from fecal samples of both species using a TGuide S96 Magnetic Soil/Stool DNA Kit (Tiangen Biotech Co., Ltd., Beijing, China), following the manufacturer’s standard protocol. High-fidelity enzymes were employed for amplification, and the sequencing Q30 value exceeded 90%. Following genomic DNA extraction from samples of both species, the V3–V4 hypervariable region of the bacterial 16S rRNA gene was amplified using the primer pair 338F (5′-ACTCCTACGGGAGGCAGCA-3′) and 806R (5′-GGACTACHVGGGTWTCTAAT-3′). After nucleic acid concentration quantification, the integrity of PCR products was verified via agarose gel electrophoresis, and target amplicons were purified using the Omega DNA Purification Kit (Omega Inc., Norcross, GA, USA). After a second round of quantification, purified amplicons from all samples were pooled in equimolar amounts to ensure uniform sequencing depth across libraries. An Illumina NovaSeq 6000 (Illumina Inc., San Diego, CA, USA) was used to sequence the constructed library. The raw data have been deposited in the NCBI Sequence Read Archive database (Accession number: PRJNA1440130 (FMD) and PRJNA1146191 (from SAMN43088433 to SAMN43088444) (AMD)). DNA extraction blanks and no-template PCR controls were included in every batch of DNA extraction and PCR amplification, respectively. All negative controls showed no detectable amplification, and no control-derived sequences were present in the final dataset after quality filtering, indicating negligible contamination.

### 2.4. Bioinformatic Analysis and Statistical Analysis

Bioinformatics analysis was performed on the BMK-Cloud platform (Biomarker Technologies Co., Ltd., Beijing, China). Raw reads were quality-filtered using Trimmomatic (v0.33) according to base quality scores. Paired-end reads were then merged using USEARCH (version 10.0), and chimeras were removed using UCHIME (version 10.0). The merged sequences were clustered into operational taxonomic units (OTUs) at 97% sequence similarity using USEARCH (version 10.0), and OTUs represented by fewer than two reads were discarded. Taxonomic annotation of the OTUs was performed using the naïve Bayes classifier in QIIME2 (version 2020.6.0) using the SILVA database (release 138, http://www.arbsilva.de (accessed on 15 November 2024)).

All downstream analyses were performed on a quality-filtered, rarefied OTU abundance table generated from 21 qualified fecal samples. Alpha and beta diversity metrics were calculated using QIIME 2. Alpha diversity was characterized by Chao 1, ACE, Shannon, Simpson, Good’s Coverage, and Faith’s Phylogenetic Diversity. The inter-group difference test was conducted using Student’s *t*-test. *p* < 0.05 is considered significant. Beta diversity was assessed via principal coordinates analysis (PCoA) and non-metric multidimensional scaling (NMDS). PCoA and NMDS were performed based on Bray–Curtis dissimilarity matrices to assess inter-group differences in gut microbial community composition between the two musk deer groups. All figures, including taxonomic composition bar plots at different taxonomic levels, NMDS ordination plots, and ANOSIM results, were generated using the ggplot2 package in R. Clustering analysis of the dataset was performed using the unweighted pair-group method with arithmetic mean (UPGMA) implemented in R. Linear discriminant analysis effect size (LEfSe) was applied to identify statistically significant differentially abundant microbial taxa between samples from the two musk deer species.

## 3. Results

### 3.1. Sequencing Quality Assessment for Wild Alpine Musk Deer and Ex Situ Conserved Forest Musk Deer

**Figure 1:**
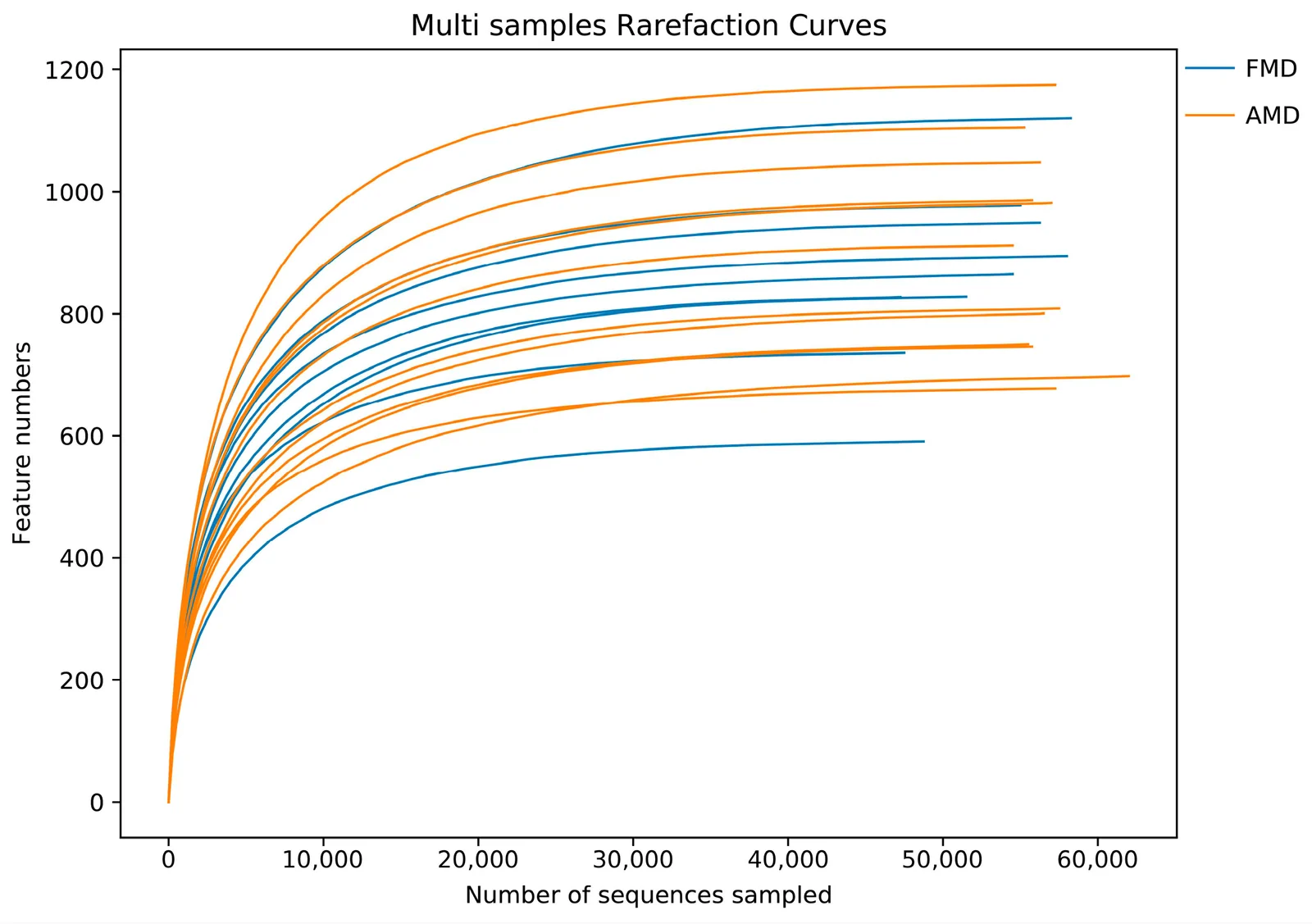
Sequencing depth rarefaction curves for wild alpine musk deer and ex situ conserved forest musk deer samples.

**Figure 2:**
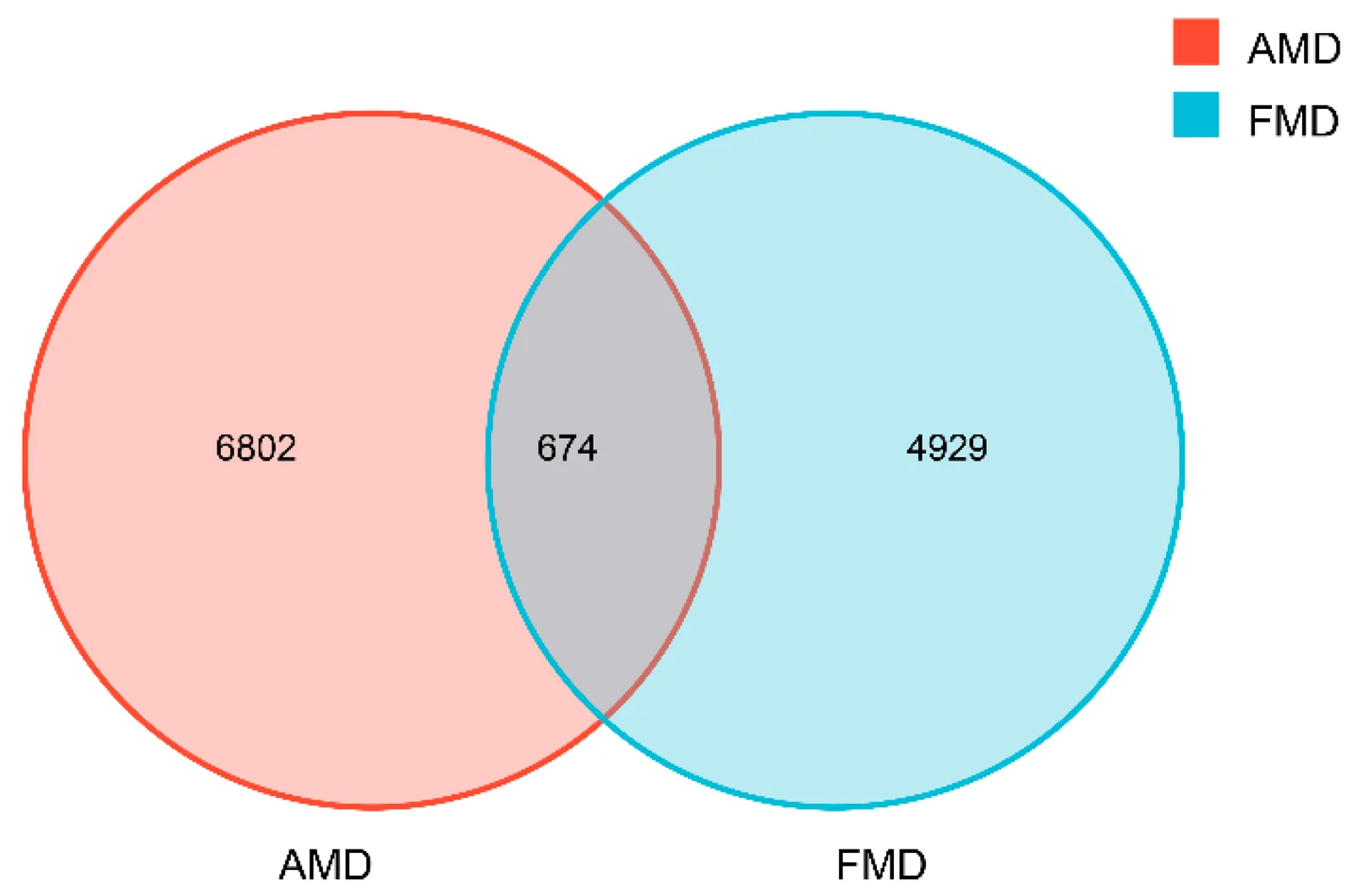
Venn diagram of gut microbiota OTUs from wild alpine musk deer and ex situ conserved forest musk deer.

A total of 1,678,492 raw paired-end reads were generated from 21 samples and 1,175,013 valid sequences were retained after quality filtering and chimera removal. Of these, 690,242 sequences were from wild AMD and 484,861 from ex situ conservation FMD. Rarefaction curves plateaued for all samples, indicating that sequencing depth was sufficient (Figure 1). Good’ s coverage exceeded 99% for all samples of both species. After removing low-abundance spurious OTUs, the remaining sequences were clustered into OTUs at a 97% sequence similarity threshold and subjected to taxonomic annotation. This yielded 12,405 OTUs in total: 7476 in the AMD group and 5603 in the FMD group. A total of 674 OTUs were shared between the two groups, accounting for 9.02% and 12.03% of the total OTUs in the AMD and FMD group, respectively (Figure 2).

### 3.2. Comparison of Gut Microbiota Composition Between Wild Alpine Musk Deer and Ex Situ Conserved Forest Musk Deer

#### 3.2.1. Gut Microbiota Diversity Analysis of Wild Alpine Musk Deer and Ex Situ Conserved Forest Musk Deer

**Figure 3:**
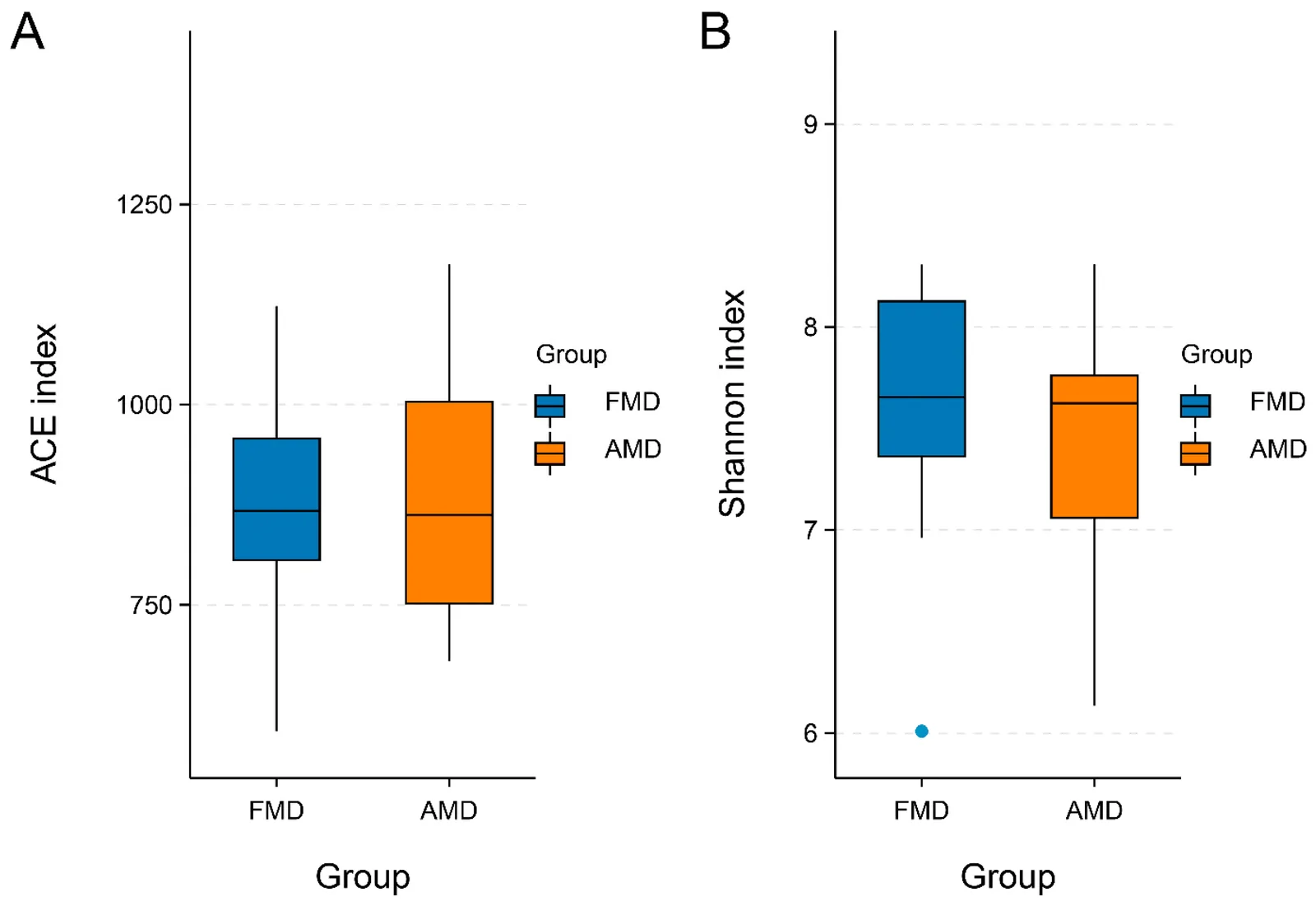
Differences in selected gut microbiota alpha diversity indices between wild alpine musk deer and ex situ conserved forest musk deer samples. (**A**) ACE index; (**B**) Shannon index.

Alpha diversity was quantified using multiple indices (e.g., ACE, Shannon index) (Figure 3). None of these indices differed significantly between wild-caught AMD and ex situ FMD (*p* > 0.05).

**Figure 4:**
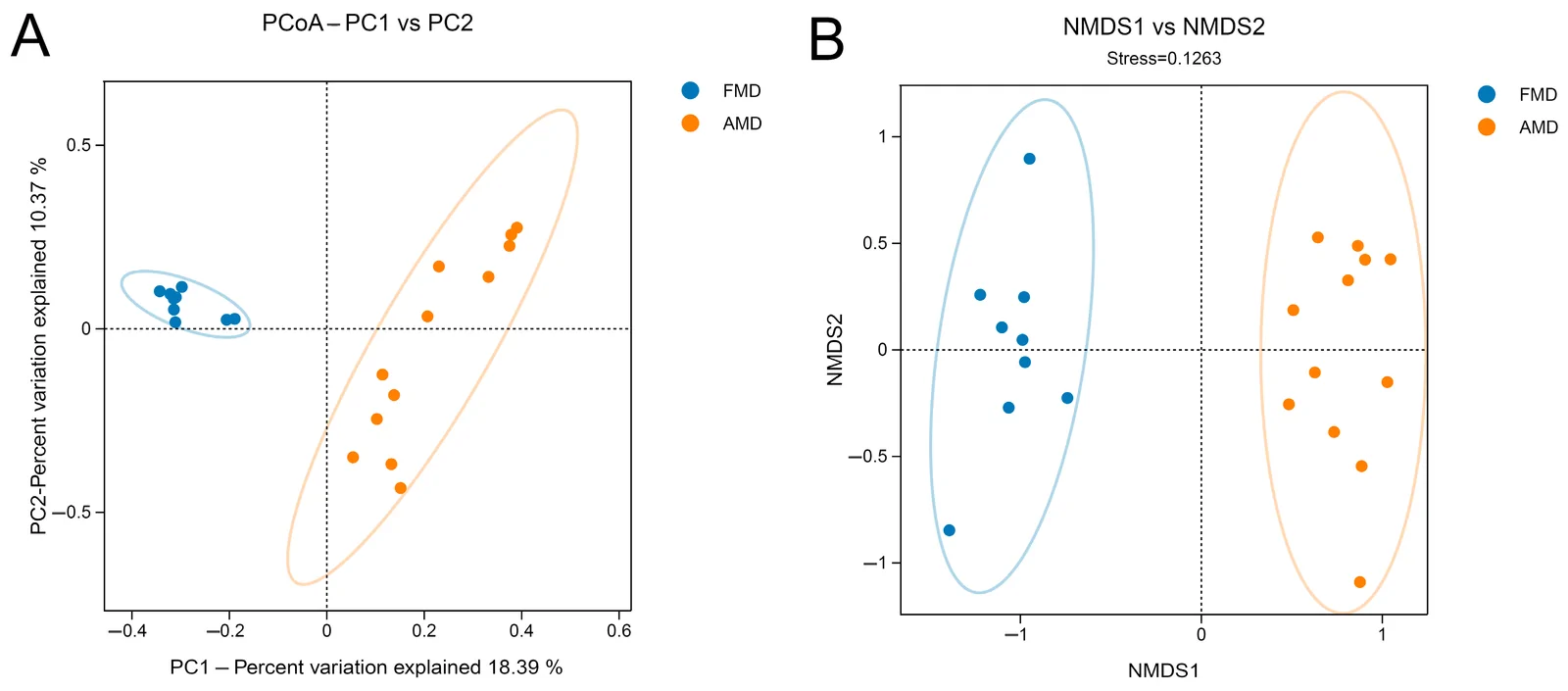
Gut microbiota beta diversity of wild alpine musk deer and ex situ conserved forest musk deer. (**A**) PCoA analysis of gut microbiota. (**B**) NMDS analysis of gut microbiota.

**Figure 5:**
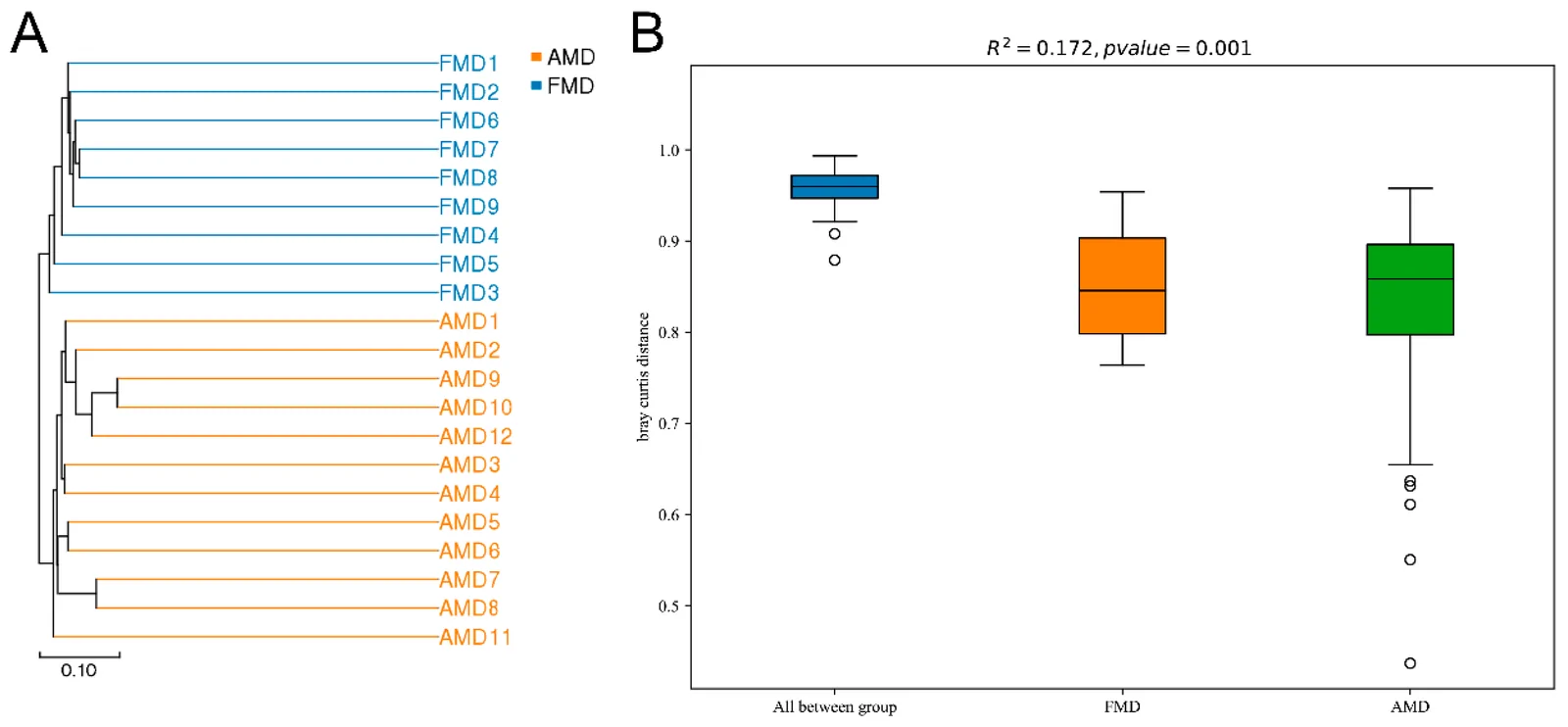
UPGMA clustering and ANOSIM analysis of gut microbiota in wild alpine musk deer and ex situ conserved forest musk deer. (**A**) UPGMA analysis of gut microbiota (**B**) ANOSIM test for interspecific differentiation of gut microbiota communities.

PCoA and NMDS were performed based on Bray–Curtis dissimilarity matrices to assess differences in gut microbial community composition between the two musk deer groups. Both analyses revealed a clear separation of gut microbial communities by host species (Figure 4), indicating that the two species harbor distinct gut microbial community structures. The NMDS ordination yielded a stress value of 0.126, below the conventional threshold of 0.2 supporting the validity of the ordination. Hierarchical clustering of gut microbial communities was performed using UPGMA. The resulting dendrogram grouped all samples into two species-specific clades (Figure 5A). ANOSIM further confirmed significant differences in gut microbiota composition between the two species (*R* > 0, *p* < 0.01; Figure 5B).

#### 3.2.2. Gut Microbiota Composition and Community Similarity and Dissimilarity

**Figure 6:**
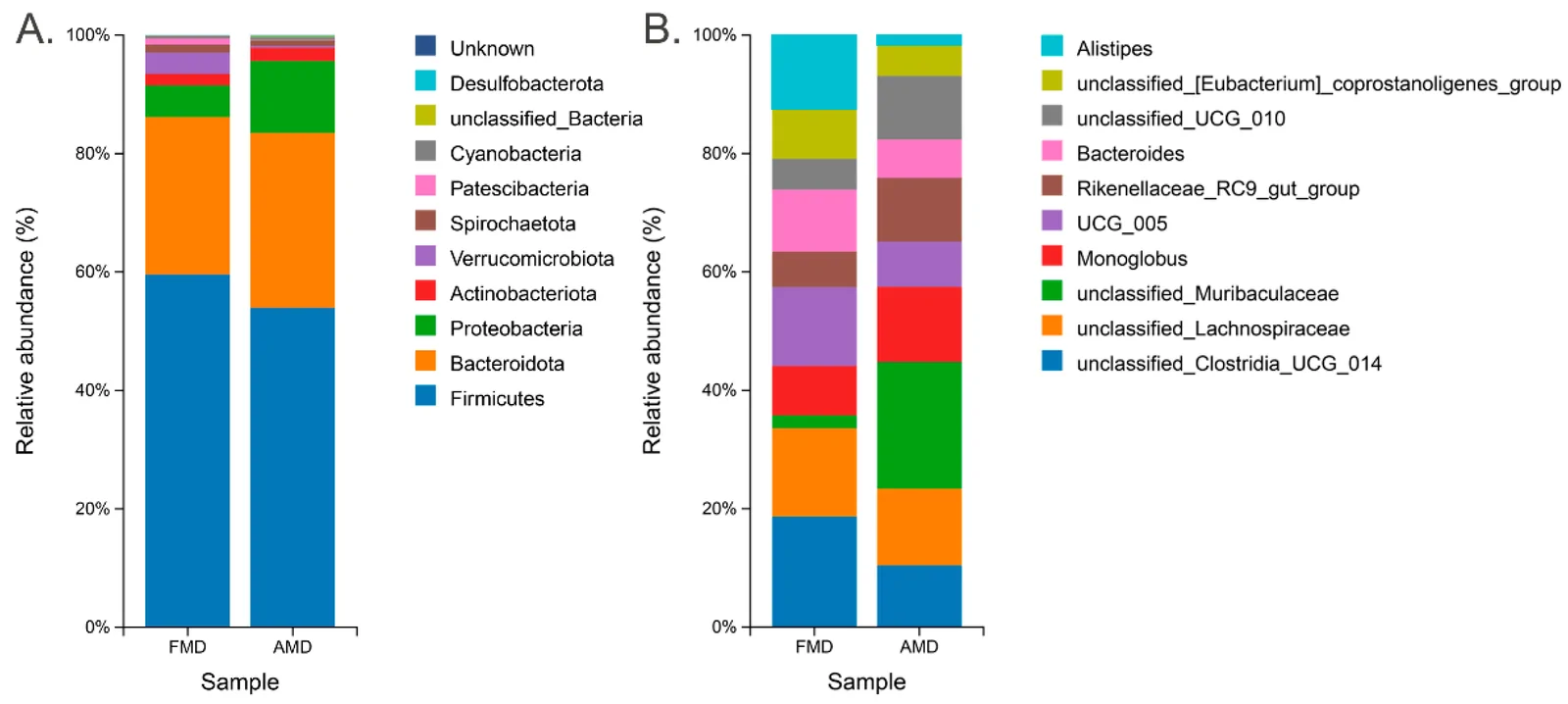
Stacked bar plots of gut bacterial microbiota composition in wild AMD and ex situ conserved FMD. (**A**) Phylum-level taxonomic composition. (**B**) Genus-level taxonomic composition.

**Figure 7:**
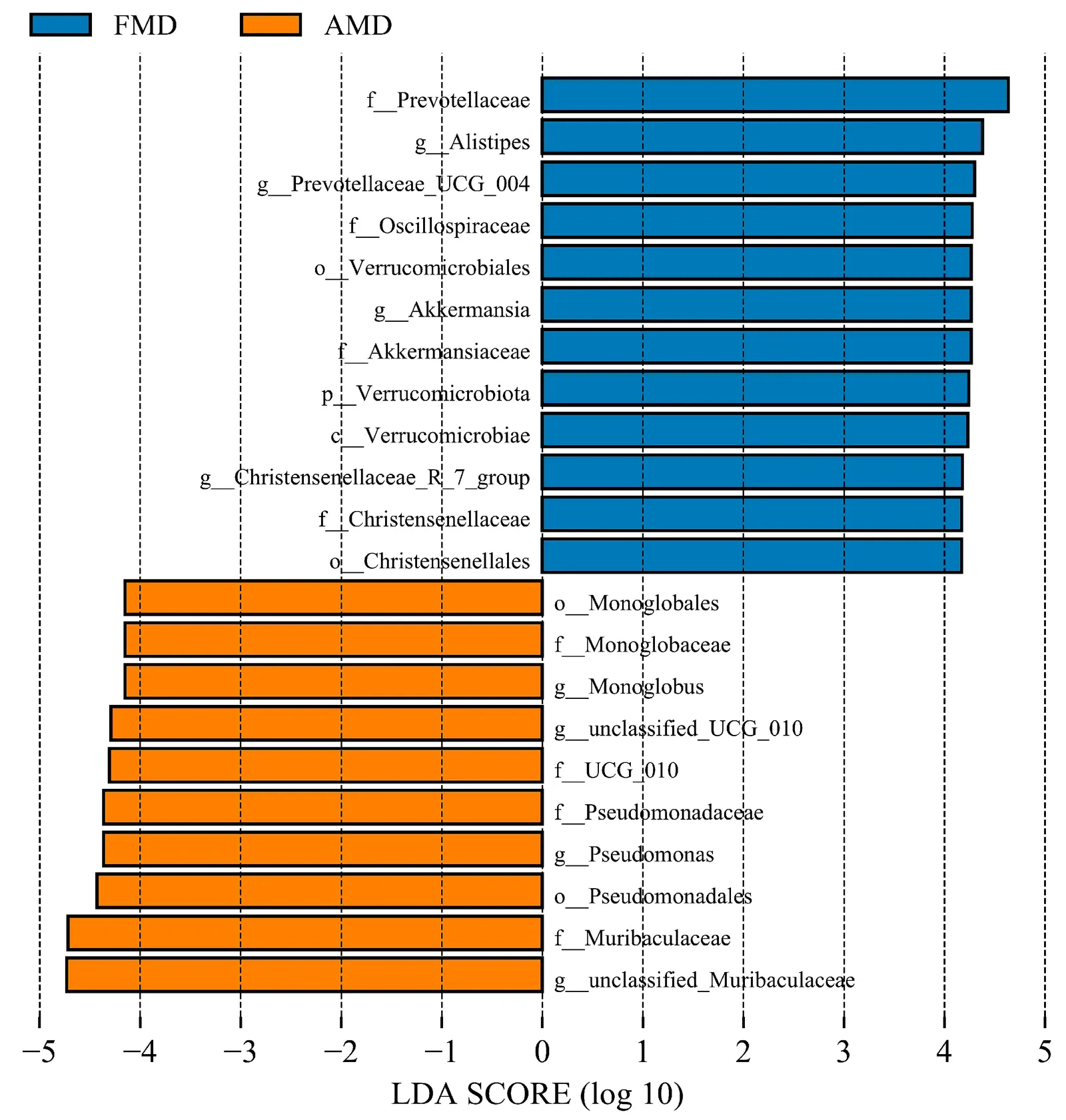
LEfSe analysis of gut microbiota between wild AMD and ex situ conserved FMD.

The 12,405 OTUs from the 21 fecal samples (wild AMD and ex situ conserved FMD) were taxonomically assigned to 35 phyla, 73 classes, 178 orders, 346 families, 714 genera, and 1197 species. At the phylum level, *Firmicutes*, *Bacteroidota*, and *Proteobacteria* were the dominant phyla in both groups (Figure 6A). The average relative abundances were 53.71%, 29.32%, and 12.12% in wild AMD, and 59.50%, 26.52%, and 5.34% in ex situ conserved FMD, respectively. Together, these three phyla accounted for over 90% of the total relative abundance in both groups. The F/B ratio was 1.83 for wild AMD and 2.24 in ex situ conserved FMD. At the family level, 346 families were identified. Among the top 10 most abundant families, the shared dominant taxa *Lachnospiraceae* and *Rikenellaceae* accounted for 11.54% and 12.72% of the community in wild AMD and 12.72% and 8.99% in ex situ conserved FMD, respectively. At the genus level, 714 genera were identified (Figure 6B). Among the top 10 most abundant genera, two shared dominant taxa were detected: unclassified genera of the family *Lachnospiraceae*, and the genus Clostridia_UCG_005, with relative abundances of 6.89% and 4.05% in wild AMD, and 6.57% and 5.85% in FMD, respectively. LEfSe analysis (LDA score > 4.0) identified differentially abundant gut bacterial taxa between the groups. These biomarkers are shown in Figure 7. The following taxa were significantly more abundant in wild AMD: the family *Muribaculaceae* and its unclassified genus, the genus *Monoglobus* (order *Monoglobales*, family *Monoglobaceae*), *Pseudomonas* (order *Pseudomonadales*, family *Pseudomonadaceae*), and unclassified genera of Clostridia_UCG_010. In contrast, the following taxa were significantly more abundant in ex situ conserved FMD: Prevotellaceae_UCG_004, *Alistipes*, Christensenellaceae_R-7_group (order *Christensenellales*, family *Christensenellaceae*), the family *Oscillospiraceae*, and *Akkermansia* (order *Verrucomicrobiales*, family *Akkermansiaceae*).

## 4. Discussion

Gut microbiota diversity is a critical determinant of host health, as it modulates digestive function and nutrient assimilation, immune capacity and disease resistance [4,17]. Higher microbial diversity confers greater functional stability, which enhances host resistance to perturbations and supports host health homeostasis [12,18]. Previous studies have documented significant differences in gut microbiota diversity across geographically isolated populations of both FMD and AMD [15]. Here, we compared the gut microbiota of wild AMD and ex situ conserved FMD that had been maintained at high altitude for over four years. No significant differences in alpha diversity indices were detected between the two groups. The comparable alpha diversity between the two groups is consistent with a degree of microbial acclimatization of ex situ conserved FMD to high-altitude conditions, using wild AMD—inhabiting similar high-altitude habitats—as the reference. Phylum-level comparison revealed no significant differences in bacterial community composition between the two groups, indicating a high degree of phylum-level similarity. This pattern is consistent with their congeneric taxonomic status and browsing feeding ecology, reflecting conserved gut microbial ecological traits across the two *Moschus* species.

At the genus level, unclassified *Lachnospiraceae* and Rikenellaceae UCG 005 dominated both wild AMD and ex situ conserved FMD, whereas *Monoglobus*, *Pseudomonas* and unclassified UCG 010 were the primary differentially abundant genera. Existing studies offer support for interpreting the observed microbial patterns: First, *Moschidae* and *Bovidae* are closely related families [19]. Intraspecific comparisons in bovids have shown that captive versus wild status has no significant effect on gut microbial community structure [20]. Further, a large-scale survey of 35 ruminant species across six continents showed that microbial community composition is driven primarily by diet, whereas host species identity has a much weaker effect [21]. Specifically, variation partitioning analysis in domestic cattle attributed up to 32.49% of gut microbial beta diversity to diet [22]. Collectively, diet is regarded as a key factor shaping the gut microbiota of herbivores [10,23], a view consistent with our finding that dietary divergence may underlie the microbial differentiation between the two musk deer populations. The ex situ conserved FMD were fed mixed dried foliage of eight plant species, supplemented with succulent feeds (carrot, white radish, pumpkin, apple and zucchini, with a small amount of concentrate mainly consisting of soybean, peanut and maize) and a small amount of concentrate (soybean, peanut, and maize). In contrast, the diet of local wild AMD is reported to comprise plant taxa from 74 families and over 140 genera [24]. The marked dietary divergence between the two populations may be the primary driver of the observed microbial divergence. The ex situ conserved FMD showed partial overlap in gut microbial taxonomic composition and inferred functional profiles with wild AMD. This convergence is consistent with the hypothesis that similar environmental pressures shape the gut microbiota of ungulates [25,26].

The gut microbial communities of wild AMD and ex situ conserved FMD were consistent with those of other ruminants in taxonomic compositional, with *Firmicutes* and *Bacteroidetes* as the dominant phyla [27]. Consistent with our findings, previous studies on the two *Moschus* species also reported *Firmicutes* and *Bacteroidetes* as the dominant phyla [28]. *Firmicutes* primarily degrade cellulose into volatile fatty acids usable by the host. *Bacteroidetes* specialize in carbohydrates (particularly polysaccharides), proteins, and other organic substrates, thereby enhancing nutrient utilization. *Proteobacteria* is also a dominant phylum in both populations. Its abundance decreases with dietary fiber content and increases with dietary protein content [29]. *Moschus* species are selective browsers, predominantly feeding on high-protein, low-fiber young foliage. This dietary pattern may partly explain the dominance of Proteobacteria in both populations. The ratio F/B ratio is widely documented to correlate with host’s adiposity and is commonly used as its proxy. An elevated F/B ratio enhances the host’s capacity to ferment dietary polysaccharides into short-chain fatty acids (SCFAs) [30]. It also mediates host energy harvest and energy homeostasis [31]. In this study, the F/B ratio was 1.83 in wild AMD and 2.24 in ex situ conserved FMD. The value in wild AMD was numerically lower than that in the ex situ conserved FMD. This pattern is consistent with the presumably lower fiber content and higher energy density of the provisioned diet, which may facilitate host energy harvest [32]. In livestock, high-concentrate diets rich in carbohydrates drive rapid production of organic acids (mainly SCFAs) in the rumen. Although SCFAs are the primary energy source for ruminants, their excessively rapid accumulation impairs ruminal buffering and induces subacute ruminal acidosis [33]. Furthermore, such diets alter ruminal microbial structure and elevate the release of lipopolysaccharides (LPS). LPS can translocate across the gastrointestinal epithelium into systemic circulation, triggering inflammatory responses [34,35]. Long-term over-provision of high-concentrate diets can induce ruminal epithelial inflammation and apoptosis, potentially leading to systemic inflammation and more severe pathological outcomes [36]. In ex situ FMD management, concentrate inclusion is commonly increased to meet the elevated thermoregulatory energy demands of high-altitude cold environments. Notably, the FMD population in this study exhibited a higher mortality rate than conspecific populations reared at low altitudes [37,38]. We postulate that the elevated gut F/B ratio in ex situ conserved FMD likely reflects over-provision of high-concentrate diets. Excessive concentrate intake may impair immune function and induce other pathological conditions, potentially contributing to elevated mortality in ex situ conserved FMD.

Beta diversity analyses—PCoA ordination, UPGMA hierarchical clustering and LEfSe—revealed clear structural divergence in gut bacterial communities between wild AMD and ex situ conserved FMD. *Muribaculaceae*, a core member of the *Bacteroidetes*, has been documented to degrade both endogenous (host-derived mucin glycans) and exogenous (dietary fiber) polysaccharides, producing SCFAs as primary fermentation end-products [39]. *Monoglobus*, a genus affiliated with the *Ruminococcaceae* originally isolated from human feces, has been reported to degrade dietary pectin. As core commensals of the herbivore gut, members of the *Ruminococcaceae* play key roles in lignocellulose breakdown and complex carbohydrate catabolism [40]. *Pseudomonas* participates in carbohydrate metabolism and can degrade organic matter via denitrification [41]. The lower abundance of *Muribaculaceae*, *Monoglobus*, and *Pseudomonas* in FMD suggests a potentially reduced capacity for dietary fiber degradation, indicating that fiber provision in the diet of ex situ conserved FMD should be increased. *Prevotella* is a dominant genus in gut microbiota of livestock. Genomic analyses indicate broad functional capacities in this genus, including core metabolism, genetic information processing, and membrane transport—traits that may reflect adaptation to niches where carbohydrates and free amino acids are limiting [42]. *Alistipes* is closely associated with lipid metabolism and has been reported to participate in its regulation [43,44]. The Christensenellaceae R-7 group, a core taxon in ruminant gut microbiota can secrete glycosidases that promote cellulose and hemicellulose degradation and produce SCFAs (e.g., acetate and butyrate) via fiber fermentation [45]. *Oscillospira* (*Firmicutes*) can produce glycoside hydrolases essential for degrading plant cell wall cellulose [21]. *Akkermansia* has been reported to alleviate intestinal inflammation and support intestinal barrier integrity, thereby modulating host immunity [46]. Comparative analysis yielded mixed signals regarding the fiber-degrading potential of the two populations. The higher abundances of the Christensenellaceae R-7 group and Oscillospira in FMD—both taxa with reported potential to degrade plant structural carbohydrates—represent one such signal. However, the concurrent enrichment of *Prevotella*, which thrives in niches with limited carbohydrates, suggests that the fiber-degrading potential of ex situ FMD may be constrained. In addition, *Alistipes* was significantly more abundant in ex situ conserved FMD than in wild AMD. This is consistent with its reported association with high-fat diets and may reflect the high-fat concentrate included in the FMD feeding regime. We also observed a higher *Akkermansia* abundance in ex situ conserved FMD. This enrichment may represent a compensatory response to maintain intestinal barrier integrity; if so, it would suggest that ex situ conserved FMD face a potentially elevated risk of intestinal inflammation.

The eastern margin of the QTP lies at the junction of China’s first and second topographic steps, forming an altitudinal transition between mid-low and high elevations. The region has a highly dissected terrain of alternating mountains and valleys, abundant precipitation, and well-developed forests. It is highly vulnerable to soil and water erosion, forms a key component of the QTP Ecological Barrier in China’s national ecological conservation framework, and is recognized as an ecologically fragile area [47,48]. Large-scale agricultural and forestry are unsuitable here. The region is also the native range of several musk deer species including FMD, AMD and black musk deer [49]. Ex situ FMD farming thus represents an important model for local under-forest ecological economies: it can provide source populations for reintroduction and sustainably supply raw musk for traditional medicine.

China launched ex situ conservation-oriented artificial breeding of FMD in 1958 and has since accumulated extensive experience and technical systems [50,51]. Ex situ conservation is widely recognized as a core strategy for mitigating extinction risk of rare and endangered fauna. Gut microbiota composition is a well-established, non-invasive biomarker for animal health; regular monitoring is therefore recommended throughout the ex situ conservation management cycle. Drawing on our findings, we propose that mitigating the elevated mortality observed during acclimatization of ex situ conserved FMD depends primarily on optimizing dietary formulation, using the natural diet and gut microbiota of wild AMD as a benchmark. The goal of this adjustment is to promote convergence of the FMD gut microbiota toward that of wild AMD at the family and genus levels. In parallel, taxa with well-characterized physiological functions should be monitored throughout the adjustment process. In addition, high-altitude regions have a much shorter development history. The study area hosts the first high-altitude ex situ FMD breeding base, established in 2018. Despite preliminary success, it still faces a high incidence of disease, especially intestinal and metabolic disorders. The breeding base lies within the range of wild AMD, whose diet and associated gut microbiota reflect long-term adaptation to the local high-altitude environment. AMD and FMD are closely related congeneric species. Therefore, for ex situ FMD populations reared in high-altitude regions, the dietary composition and gut microbial community structure of wild AMD should be used as a baseline reference to enhance their high-altitude acclimatization capacity.

## 5. Conclusions

This study compared the gut microbiota of wild AMD and ex situ conserved FMD. No significant differences in alpha diversity were detected in the two groups, whereas their community structures differed significantly. The F/B ratio was higher in FMD than in AMD. This pattern is consistent with the presumably lower fiber content and higher energy density of the provisioned diet, which may facilitate host energy intake. The divergence in community structure may be driven primarily by dietary differences between the two populations. Based on the above findings, first, we propose that feeding protocols for ex situ conserved FMD should be optimized using the natural forage of wild AMD as a benchmark. Second, further research should examine how these forage species shape the gut microbiota of both species. Such efforts would advance high-altitude ex situ breeding of FMD and contribute to the conservation of this endangered species.

## Data Availability

The raw amplicon and metagenome sequences were deposited under the NCBI BioProjects: PRJNA1440130 and PRJNA1146191 (from SAMN43088433 to SAMN43088444).

## Abbreviations

AMD	Alpine musk deer
FMD	Forest musk deer
F/B	*Firmicutes*/*Bacteroidetes*
QTP	Qinghai–Tibet Plateau
OTU	Operational taxonomic unit
PCoA	Principal coordinate analysis
NMDS	Non-metric multidimensional scaling
ANOSIM	Analysis of similarities
UPGMA	Unweighted pair-group method with arithmetic mean
LEfSe	Linear discriminant analysis effect size
SCFAs	Short-chain fatty acids
LPS	Lipopolysaccharides
